# Oral Health of Elderly People in Institutionalized Care and Three-Month Rehabilitation Programme in Southern Poland: A Case-Control Study

**DOI:** 10.3390/ijerph19094994

**Published:** 2022-04-20

**Authors:** Piotr Michalak, Paulina Polak-Szlósarczyk, Wioletta Dyduch-Dudek, Elżbieta Zarzecka-Francica, Maria Styrna, Łukasz Czekaj, Joanna Zarzecka

**Affiliations:** 1 Department of Conservative Dentistry with Endodontics, Institute of Stomatology, Faculty of Medicine, Jagiellonian University Medical College, 31008 Krakow, Poland; paulina.polak@poczta.fm (P.P.-S.); wioletta.dyduch@interia.pl (W.D.-D.); lukaszcz89@gmail.com (Ł.C.); j.zarzecka@uj.edu.pl (J.Z.); 2 Department of Prosthetics, Institute of Stomatology, Faculty of Medicine, Jagiellonian University Medical College, 31008 Krakow, Poland; elzbieta.zarzecka@uj.edu.pl; 3 Municipal Health Centre for Older and Dependent Individuals, 30663 Krakow, Poland; maria.styrna@gmail.com

**Keywords:** older people, gerodontology, oral health, dry mouth, long-term care

## Abstract

Demographic ageing is a global growing process and the quality of ageing is an important parameter in this process. The aim of the study was to analyse the distribution of remaining dentition in relation to oral hygiene indicators among elderly people remaining in institutional care and those who participated in a 3-month rehabilitation program, aimed at increasing time of independent functioning, in southern Poland. The patients underwent a questionnaire and clinical examination. An analysis of missing teeth was performed, plaque index, and gingival index were measured. Residents of the Municipal Health Centre for Older and Dependent People (*n* = 50) had a higher incidence of missing teeth in the maxilla (88.4%), mandible (77.6%), as well as in the maxilla and mandible combined (83%) than residents of the Daily Medical Care House (*n* = 30). The distribution of the remaining teeth, in both groups, corresponds to the outlets of the large salivary glands. The group of 53.8% of patients with dry mouth had PI scored 3. Residents with dry mouth were more likely to have plaque deposits and gingival inflammation. It is necessary to develop and implement an oral care program for patients with reduced saliva secretion, with a particular focus on dependents.

## 1. Introduction

Due to the demographic changes observed in terms of a longer life expectancy. several new challenges for health services have emerged [1,2].

In 2014, the term “new potential geriatric syndrome” was proposed for the oral health of the elderly. This means that geriatric syndromes (GS) such as frailty, falls, functional limitations, depression, malnutrition, polypharmacy, and cognitive impairment are considered to include oral health that deteriorates with age [3].

Good oral health is important at any time of life, mainly due to its functions and the possibility of primary inflammatory foci. Primary foci of infection are chronic inflammatory conditions with the potential for harmful effects in distant tissues or organs. In the oral cavity these are mainly: dental caries, residual roots, chronic inflammation of periapical tissues, oral mucosa and periodontitis [4,5,6]. Poor oral hygiene promotes their formation.

In old age, maintaining good oral hygiene can be difficult [7]. An important factor that contributes to poor hygiene is a disorder of saliva secretion. It is most often a side effect of medications taken or a consequence of therapeutic irradiation [8,9]. The change in dietary habits often observed in old age favors the maintenance of the metabolism of dental plaque [10].

In addition to local factors, general health, aforementioned multimorbidity, multidrug use and possible cognitive impairment increase the risk of poor hygiene and the development of plaque-related conditions [11].

Regular and professional removal of pathological dental deposits and preventative procedures play an important role in maintaining good oral hygiene and health. For this reason, the independence of older people and easy access to a dentist are important. 

In this study, we assumed that the degree of independence of the older people surveyed would have an impact on oral conditions. Taking into account the specificity of the care organization for the elderly in different countries or even regions of a single country, we wanted to learn about the specificity of the tested group, the inhabitants of Malopolska.

The aim was to analyze distribution of remaining dentition in relation to oral hygiene indicators among elderly people remaining in institutionalized care and those who participated in a 3-month rehabilitation program to increase the time of independent functioning in southern Poland. 

## 2. Materials and Methods

The study involved 80 patients, 50 residents of a Municipal Health Centre for Older and Dependent Individuals (MHCOD) and 30 participants of a 3-month rehabilitation program at a Daily Medical Care House (DMCH). During a 3-month course, there were 15 residents of DMCH. MHCOD is an independent public health care institution supervised by the Health Office of the City of Krakow. DMCH is a form of activity of this facility that aims to extend the period of independent living for older people. In the MHCOD group, patients received 24-h care while participants in the 3-month rehabilitation stay lived independently or with their families. The evaluation of the cognitive abilities of the patients who participated in the study was carried out by a psychologist using the MMSE scale (Mini-Mental State Examination). MMSE was assessed in the following categories: normal >25 points, suspected cognitive impairment between 21 and 24 points, and severe cognitive impairment < 20 points. Patients who agreed to participate in the study signed an informed consent. The group examined by the psychologist did not show significant cognitive impairment.

Patients were subjected to a questionnaire and an intraoral clinical examination. The questionnaires included questions about the symptoms reported most frequently by senior patients in clinical practice. Due to the lack of medical knowledge among patients, these were questions about subjective sensations. Based on the symptoms reported, healthcare providers and dentists can perform additional diagnostics to confirm possible conditions. In the questionnaire the participants answered questions about pain in the mouth, bleeding gums, tooth mobility, bad breath, burning mucosa, excess saliva, or dryness, swallowing difficulties, pain in the temporomandibular joint. Questions related to hygiene habits were also asked: self-efficacy in performing hygiene tasks, use of additional oral hygiene utensils, mouthwash and frequency of cleaning teeth and dentures. For each question the respondent could answer yes or no.

The clinical examination was preceded by the investigator calibration and was performed by two dentists in a medical office setting, under headlight, using a dental mirror and a dental probe [12]. An analysis of missing teeth was performed (FDI, 1970). Plaque Index (PI Silness & Löe 1964) and Gingival Index (GI, Löe & Silness 1963) were measured. Third morlars were excluded from the calculation.

Quantitative characteristics were described by the arithmetic mean, median, minimum, maximum and standard deviation. Qualitative characteristics were described by the number of teeth per group (*n*) and percentage (%), then tabulated in multivariate tables. Differences in quantitative characteristics between two groups were evaluated using the Student’s *t*-test, or the Mann-Whitney U test, depending on the distribution of the characteristics. The evaluation of differences and dependencies of qualitative traits was verified by the chi-square test or Fisher’s exact test. Spearman’s order correlation (rs) was used to assess the association of quantitative characteristics (PI and GI). Statistical calculations were performed with the STATISTICA 13 program.

The study was carried out with the approval of the Jagiellonian University Bioethics Committee 1072.6120.187.2017.

## 3. Results

### 3.1. Group Characteristic

Among the MHCOD patients, there were 31 women (62%) and 19 men (38%), while in DMCH women were 21 (70%) and men were 9 (30%). There were no statistically significant differences in the incidence of women and men between MHCOD and DMCH (*p* = 0.467) (Table 1).

The mean age for women in MHCOD was 78.2 years and for DMCH 72.9 years, while for men these values were 66.5 and 70.6 years, respectively. The analysis did not show a correlation within age between the women (*p* = 0.222) and men (*p* = 0.657) groups in MHCOD and DMCH (Table 1).

The comparison of patients who remained in MHCOD and DMCH care using MMSE did not show statistically significant relationship (median 26.0; *p* = 0.360; mean MHCOD = 25.0; mean DMCH = 25.8) (Table 2). Such results provided an opportunity to compare two groups.

### 3.2. Oral Health Conditions

Among MHCOD residents, the number of toothless people was 25; among the DMCH rehabilitation program, the number was 11. This means that among MHCOD residents the number of people with their own teeth was 25 and in the DMCH group 19. 

In the analysis of missing teeth patients with edentulism and having their own teeth were taken into account.

In the analysis of the number of missing molars there were significantly more missing molars among MHCOD patients than in DMCH patients, both in the maxilla, mandible and in the total maxilla and mandible (*p* < 0.001). Mandibular molars were missing more frequently than maxillary, among residents of MHCOD (*p* = 0.015) and DMCH (*p* = 0.049). Molars were the most frequently missing among residents of MHCOD in the mandible (96.5%) and the least among DMCH in the maxilla (75.8%) (Table 3).

Similarly mong premolars, more missing teeth were found in MHCOD than in DMCH in the maxilla, mandible as well as in the maxilla and mandible in total (*p* < 0.001; *p* = 0.015 and *p* < 0.001, respectively). Compared to molars, premolars were missing more frequently in the maxilla (MHCOD *p* < 0.001 and DMCH *p* = 0.035). The premolars were the most frequently missing among MHCOD residents in the maxilla (90.5%) and the least among the DMCH group in the mandible (63.3%) (Table 3).

In the analysis of missing canines similarly to molars and premolars, we found that canines were missing more frequently in the MHCOD resident group in the maxilla, mandible as well as in the maxilla and mandible in total (*p* < 0.001; *p* = 0.011; *p* < 0.001, respectively). As with premolars, canines were more frequently missing in the maxilla (MHCOD *p* < 0.001; DMCH *p* = 0.017). Canines were mostly missing in the MHCOD resident group in the maxilla (88.0%), and the least frequently among DMCH in the mandible (43.3%) (Table 3).

According to missing incisors, our studies showed that more missing teeth were observed among MHCOD residents than DMCH in the maxilla, mandible and in the maxilla and mandible in total (*p* < 0.001; *p* = 0.010; *p* < 0.001, respectively). As in premolars and canines, the incisors were missing more frequently in the maxilla (MHCOD *p* < 0.001; DMCH *p* = 0.012). Incisors were missing most of the time in the MHCOD residents’ group in the maxilla (84.5%) and the least frequently among DMCH in the mandible (52.5%) (Table 3).

**Figure 1:**
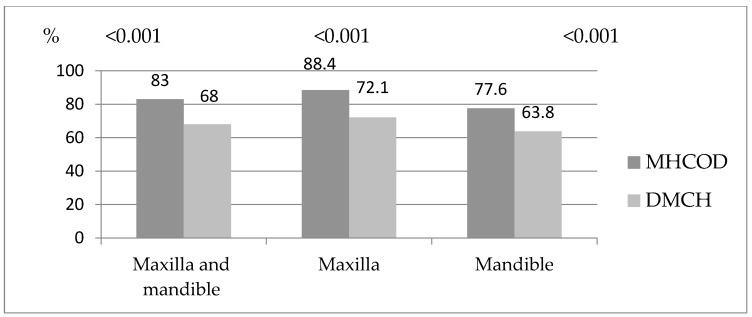
Distribution of missing teeth between the study groups.

To summarize, our studies showed that MHCOD residents had a higher incidence of missing teeth in the maxilla, mandible, as well as in the maxilla and mandible combined (*p* < 0.001). Among the studied groups there were more missing teeth in the maxilla than in the mandible (Figure 1).

Patients with their own dentition (at least 1 tooth) were considered for PI and GI assessment. This was a group of 44 patients, including 25 MHCOD and 19 residents of DMCH. Statistical analysis showed a slightly higher PI score for residents of MHCOD compared to DMCH, with medians reaching 2 and 1, respectively (*p* = 0.160). There were no statistically significant differences between GI for the study groups (Table 4).

**Figure 2:**
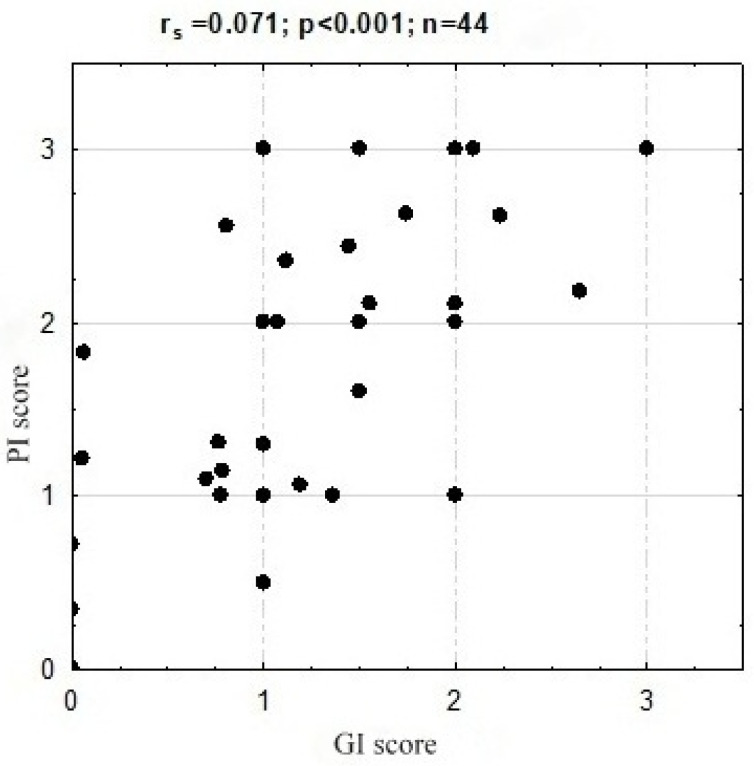
Spearman rank order correlation.

Spearman rank order correlation analysis showed a high correlation of PI and GI (r_s_ = 0.071 *p* < 0.001) (Figure 2).

**Figure 3:**
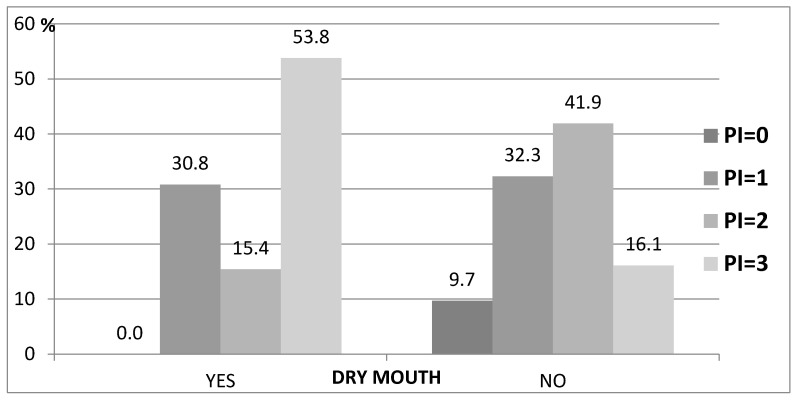
Relationship between PI and dry mouth.

An analysis of the results of the questionnaire showed the presence of a relationship between PI, dry mouth and self-care. Among 53.8% of patients with dry mouth the PI value was 3, compared to patients who did not complain of dry mouth, where this result occurred in only 16.1% (*p* = 0.047) (Table 5, Figure 3).

**Figure 4:**
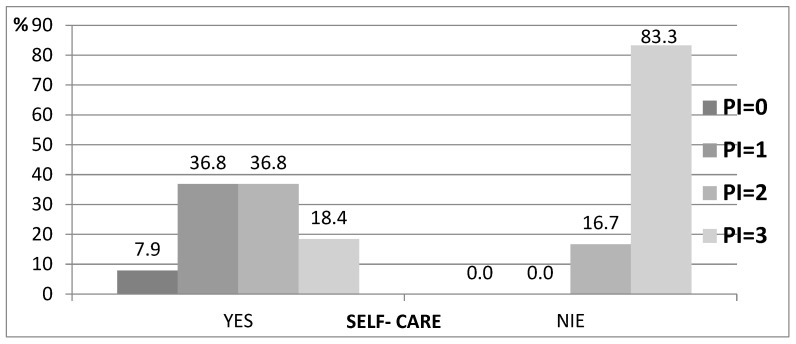
Relationship between PI and self-care.

A significant influence of the ability to independently take care of proper oral hygiene on the value of PI and GI was found. Only 18.4% of the residents who performed their own hygiene activities had a PI = 3, while 83.3% of the dependents had a PI = 3 (*p* = 0.010) (Table 5, Figure 4).

**Figure 5:**
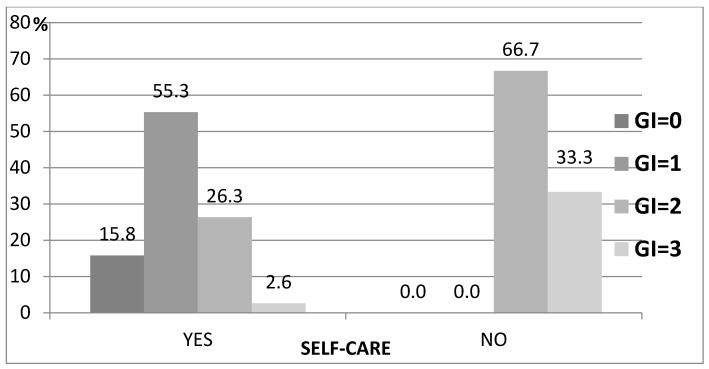
Relationship between GI and self-care.

Regarding to the gingival index, among residents performing hygienic activities on their own, 26.3% had GI = 2 and 2.6% subjects had GI = 3. In the group of residents whose oral hygiene was dependent on carers, as many as 66.7% had GI = 2 and 33.3% GI = 3 (*p* = 0.003) (Table 6, Figure 5).

An analysis of the relationship between the frequency of cleaning teeth and dentures in the study groups showed the presence of a statistically significant relationship (*p* = 0.033). In the MHCOD residents’ group, up to 40% admitted to oral hygiene once every two days, while in DMCH only 16.7% of respondents. However, a higher frequency, several times a day, was reported by 20% and 50%, respectively (Table 7).

## 4. Discussion

Demographic ageing is a global and growing process, and the quality of ageing is an important factor in this process. As the number of seniors in the general population increases, the proportion of people 65 years and older in Poland reaching 18% in 2019, adequate care for the ageing population is becoming more important [13]. 

Seniors face many challenges. These include multimorbidity, exclusion (“ageism”), poverty, loneliness, and dependence on carers for basic life activities [3,14,15,16]. Senior citizens’ centers address these issues. In Poland, the main sources of assistance for older people are the Municipal Health Centre for Older and Dependent Individuals (MHCOD) and the Daily Medical Care House (DMCH). In many countries, including Poland, there are no established standards of practice for the care of oral health and hygiene among residents of long-term residential care [17].

Our study included 80 patients. Among them were 50 MHCOD and 30 residents of DMCH. The analysis showed that the main group of people within both MHCOD and DMCH was women, 62% and 70%, respectively. The mean age of women in the MHCOD and DMCH groups was higher than men (78.2 years and 72.9 years for women, 66.5 years and 70.6 years for men, respectively). The results obtained are in line with studies conducted by other authors [17,18,19,20,21,22,23]. This may be due to the average life expectancy, which in 2030 in Poland is expected to be 84 years for women compared to 77.3 years for men [13].

We observed that in the study groups, the most frequent missing teeth were molars followed by premolars. The frequency of missing teeth of each group is higher in MHCOD than in DMCH patients, in both maxilla and mandible. This is probably due to the poorer general health condition and greater dependence of nursing home patients on carers. Such assumptions are consistent with studies by other authors which show that seniors who need assistance with oral hygiene have more decayed teeth, fewer fillings, resulting in multiple missing teeth [18]. With a high frequency of missing premolars, a lack of occlusal contacts creates a risk of nutritional disorders [24]. The prevention of malnutrition is crucial for seniors staying in long-term care. Malnutrition leads to reduced ability to perform basic life activities, lower quality of life (QoL), longer hospital stays and rehabilitation, higher risk of falls, more frequent infections, impaired wound healing and higher mortality [25,26,27,28]. Our study also showed that the number of completely edentulous seniors was higher in MHCOD than in DMCH, 50% and 36.66%, respectively. Publications by other authors that analyze the risk of edentulousness according to sex have shown that older women are at higher risk compared to their male peers [19]. A study conducted in Poland showed that 47.1% of seniors over 65 years of age are completely edentulous, and more than half are women [29]. The higher risk of edentulousness among women is most likely due to the risk of periodontitis and a higher propensity for caries. Awareness of and attention to oral hygiene mean that women receive more dental treatment, have fewer residual roots, and use dentures more often [29,30].

In our study, we performed a detailed analysis of the distribution of missing teeth. This allowed us to present statistically significant relationships between missing tooth and its location. Molars were missing more frequently in the mandible compared to the other tooth groups. The distribution of the remaining dentition coincides with the outlets of the large salivary glands. Together they are responsible for the secretion of 90% of total saliva [31]. Saliva components, both organic and inorganic, play an important role in caries prevention [32]. Elderly patients suffer from hyposalivation as a result of general illness and complex pharmacotherapy [33]. A study in the Netherlands found that among residents of nursery homes, the incidence of hyposalivation ranges from 24% for resting saliva to 60% for stimulated saliva. Additionally, the volume of saliva secreted was significantly lower in women than in men and in older people than in younger residents [34]. The negative effect of reduced salivary secretion is enhanced by a decrease in the efficiency of saliva flow and mixing within the oral cavity. It is a consequence of the progressive decrease in muscle tone of the tongue, cheeks, and lips observed with age [35,36]. The cumulative effect of these factors may explain our results, which indicated that teeth located near salivary glands’ duct are more likely to remain in the oral cavity.

The analysis showed statistically significant relationships with respect to PI and GI. The high correlation between these indicators is the evidence that plaque accumulation and poor oral hygiene are risk factors for gingivitis and periodontitis also among seniors. This relationship was confirmed by many other studies [37,38]. Furthermore, we showed a correlation between PI and dry mouth. More than half of the residents who complained of dry mouth had the highest PI (53.8%, *p* = 0.047). This relationship was confirmed in studies involving ‘younger adults’, indicating that xerostomia is an indirect risk factor for gingival inflammation due to increased plaque accumulation [39].

Our research has shown that among patients who depend on the help of caregivers, oral hygiene is neglected. PI and GI scores, among the analyzed groups, showed greater plaque accumulation and risk of gingival inflammation. This may suggest a lack of training for those caring for seniors in MHCOD and DMCH or caring for them at home. Furthermore, many caregivers, including nursing staff, do not have adequate knowledge of the role of plaque and diet in the etiology of dental caries [40]. This problem is raised in many studies, mentioning the need to implement educational programs and standardize procedures [3,18,19,41,42,43]. The assessment of the ability and frequency of patients to perform hygiene procedures revealed that residents of DMCH reported significantly more frequent teeth cleaning, and this relationship was statistically significant (*p* = 0.033). This result may be related to the complexity of dental deficiencies, which more often affected residents of nursery houses. Research in this field has been carried out in Korea and has shown that oral hygiene performance is related to the degree of dependence of patients and the presence of teeth and is not affected by cognitive decline due to dementia [17].

It should be noted that the results presented in our study come from a centre with residents from the southern part of Poland. Therefore, they reflect the specificity of the region. Furthermore, the analysis was carried out in a group of only 80 people. The small number of DMHC patients is due to the fact that for a 3-month course, there were only 15 participants. In addition, older people who participated in the study had to sign an informed consent. The patients selected for the study were qualified on the basis of the MMSE score and the lack of a cognitively impaired control group is a limitation of the study. A comparison of our results with results from future studies conducted in other parts of Poland will provide more sufficient conclusions. 

## 5. Conclusions

DMCH patients brush their teeth and clean their dentures more often.

There is a higher prevalence of missing teeth among residents of MHCOD compared to DMCH. The distribution of the remaining teeth, in both groups, corresponds to the outlets of the large salivary glands.

Residents with dry mouth are more likely to have plaque deposits and gingival inflammation.

An assessment of missing teeth, interview and psychical examinations for xerostomia should be an important element of geriatric examinations. 

Further studies conducted in institutionalized long-term care facilities for older people should be based on a group of residents with salivary disorders. It will enable the development of standardized procedures, appropriate support programs, and prophylaxis for residents in long-term care facility.

## Figures and Tables

**Table 1:** Demographic data.

Demographic Data		MHCOD		DMCH		*p*
		*n*	%	*n*	%	
Sex	Women	31	62	21	70	0.467
	Men	19	38	9	30	
		mean		mean		
Age	Women	78.2		72.9		0.222
	Men	66.5		70.6		0.657

**Table 2:** Mini-Mental State Examination (MMSE).

Group	Mini-Mental State Examination					*p*
	Mean	Median	Minimum	Maksimum	Standard Deviation	
MHCOD	25	26	19	30	3.1	0.36
DMCH	25.8	26	21	30	3	

**Table 3:** Distribution of missing teeth in the study groups.

Localization	Municipal Health Centre for Older and Dependent Individuals		Daily Medical Care House		*p*
	Missing Teeth	%	Missing Teeth	%	
**Molars**					
Maxilla	181	90.5	91	75.8	<0.001
Mandible	193	96.5	103	85.8	<0.001
*p*	0.015		0.049		
Maxilla + Mandible	374	93.5	194	80.8	<0.001
**Premolars**					
Maxilla	181	90.5	91	75.8	<0.001
Mandible	152	76	76	63.3	<0.015
*p*	<0.001		0.035		
Maxilla + Mandible	333	83.3	167	69.6	<0.001
**Canines**					
Maxilla	88	88	39	65	<0.001
Mandible	64	64	26	43.3	0.011
*p*	<0.001		0.017		
Maxilla + Mandible	152	76	65	54.2	<0.001
**Incisors**					
Maxilla	169	84.5	82	68.3	<0.001
Mandible	134	67	63	52.5	0.01
*p*	<0.001		0.012		
Maxilla + Mandible	303	75.8	145	60.4	<0.001

**Table 4:** PI and GI scores in the study groups.

Index	Group	Number of Residents	Mean	Median	Minimum	Maximum	Standard Deviation	*p*
PI	MHCOD	25	2	2	0	3	1	0.06
	DMCH	19	2	1	0	3	1	
GI	MHCOD	25	1	1	0	3	1	0.297
	DMCH	19	1	1	0	2	1	

**Table 5:** Results of the questionnaire in relation to the plaque index.

Point of Quesstionaire		PI Score								In Total		*p*
		0		1		2		3				
		*n*	%	*n*	%	*n*	%	*n*	%	N	%	
Pain	YES	0	0	2	40	1	20	2	40	5	11.36	0.761
	NO	3	7.7	12	30.8	14	35.9	10	25.6	39	88.64	
Bleeding	YES	0	0	2	66.7	1	33.3	0	0	3	6.82	0.507
	NO	3	7.3	12	29.3	14	34.1	12	29.3	41	93.18	
Tooth mobility	YES	0	0	0	0	0	0	1	100	1	2.27	0.435
	NO	3	7	14	32.6	15	34.9	11	25.6	43	97.73	
Halitosis	YES	0	0	0	0	1	33.3	2	66.7	3	6.82	0.38
	NO	3	7.3	14	34.1	14	34.1	10	24.4	41	93.18	
Burning of the oral mucosa	YES	0	0	0	0	1	100	0	0	1	2.27	0.577
	NO	3	7	14	32.6	14	32.6	12	27.9	43	97.73	
Excess of saliva	YES	0	0	0	0	1	100	0	0	1	2.27	0.577
	NO	3	7	14	32.6	14	32.6	12	27.9	43	97.73	
Dry mouth	YES	0	0	4	30.8	2	15.4	7	53.8	13	29.55	0.047
	NO	3	9.7	10	32.3	13	41.9	5	16.1	31	70.45	
Swallowing malfunction	YES	0	0	2	33.3	2	33.3	2	33.3	6	13.64	0.902
	NO	3	7.9	12	31.6	13	34.2	10	26.3	38	86.36	
Pain in the temporomandibular joint	YES	0		0		0		0		0		-
	NO	3	6.8	14	31.8	15	34.1	12	27.3	44	100	
Independent performance of hygienic tasks	YES	0	0	14	36.8	14	36.8	7	18.4	38	86.36	0.01
	NO	3	7.9	0	0	1	16.7	5	83.3	6	13.64	
Other items for oral hygiene	YES	1	16.7	4	66.7	0	0	1	16.7	6	13.64	0.098
	NO	2	5.3	10	26.3	15	39.5	11	28.9	38	86.36	
Mouthwashes	YES	0	0	2	66.7	0	0	1	33.3	3	6.82	0.459
	NO	3	7.3	12	29.3	15	36.6	11	26.8	41	93.18	

**Table 6:** Results of the questionnaire in relation to the GI.

Point of Quesstionaire		GI Score								In Total		*p*
		0		1		2		3				
		*n*	%	*n*	%	N	%	*n*	%	*n*	%	
Pain	YES	0	0	2	40	3	60	0	0	5	11.36	0.452
	NO	6	15.4	19	48.8	11	28.2	3	7.7	39	88.64	
Bleeding	YES	0	0	3	100	0	0	0	0	3	6.82	0.317
	NO	6	14.6	18	43.9	14	34.1	3	7.3	41	93.18	
Tooth mobility	YES	0	0	1	100	0	0	0	0	1	2.27	0.772
	NO	6	14	20	46.5	14	32.6	3	7	43	97.73	
Halitosis	YES	0	0	2	66.7	1	33.3	0	0	3	6.82	0.824
	NO	6	14.6	19	46.3	13	31.7	3	7.3	41	93.18	
Burning of the oral mucosa	YES	0	0	0	0	1	100	0	0	1	2.27	0.533
	NO	6	14	21	48.8	13	30.2	3	7	43	97.73	
Excess of saliva	YES	0	0	0	0	1	100	0	0	1	2.27	0.533
	NO	6	14	21	48.8	13	30.2	3	7	43	97.73	
Dry mouth	YES	0	0	7	53.8	4	30.8	2	15.4	13	29.55	0.199
	NO	6	19.4	14	45.2	10	32.3	1	3.2	31	70.45	
Swallowing malfunction	YES	0	0	5	83.3	1	16.7	0	0	6	13.64	0.288
	NO	6	15.8	16	42.1	13	34.2	3	7.9	38	86.36	
Pain in the temporomandibular joint	YES	0	0	0	0	0	0	0	0	0	0	─
	NO	6	13.6	21	47.7	14	31.8	3	6.8	44	100	
Independent performance of hygienic tasks	YES	6	15.8	21	55.3	10	26.3	1	2.6	38	86.36	0.003
	NO	0	0	0	0	4	66.7	2	33.3	6	13.64	
Other items for oral hygiene	YES	2	33.3	3	50	1	16.7	0	0	6	13.64	0.4
	NO	4	10.5	18	47.4	13	34.2	3	7.9	38	86.36	
Mouthwashes	YES	0	0	2	66.7	1	33.3	0	0	3	6.82	0.824
	NO	6	14.6	19	46.3	13	31.7	3	7.3	41	93.18	

**Table 7:** Frequency of teeth and denture cleaning in study groups.

Frequency of Teeth and Denture Cleaning	Residents				In Total	*p*
	MHCOD		DMCH			
	*n*	%	*n*	%		
Not at all	1	2	0	0	1	0.033
Less than once every two days	2	4	0	0	2	
Once every two days	20	40	5	16.7	25	
Once daily	17	34	10	33.3	27	
Several times a day	10	20	15	50	25	
In total	50	100	30	100	80	-

## Data Availability

The data that support the findings of this study are available from the corresponding author (P.M.), upon reasonable request.
